# A multi-centre, randomised controlled trial of cognitive therapy to prevent harmful compliance with command hallucinations

**DOI:** 10.1186/1471-244X-11-155

**Published:** 2011-09-30

**Authors:** Max Birchwood, Emmanuelle Peters, Nicholas Tarrier, Graham Dunn, Shon Lewis, Til Wykes, Linda Davies, Helen Lester, Maria Michail

**Affiliations:** 1 School of Psychology, University of Birmingham, Edgbaston, B15 2TT, Birmingham, UK; 2 King's College London, Institute of Psychiatry, Psychology Department, De Crespigny Park, London, SE5 8AF, UK; 3 School of Community Based Medicine, University of Manchester, Oxford Road, Manchester, M13 9PT, UK; 4 Primary Care Clinical Sciences, School of Health and Population Sciences, Primary Care Clinical Sciences Building, University of Birmingham, Edgbaston, B15 2TT, Birmingham, UK; 5 NIHR Biomedical Research Centre for Mental Health, South London and Maudsley NHS Foundation Trust, UK

## Abstract

**Background:**

Command hallucinations are among the most distressing, high risk and treatment resistant symptoms for people with psychosis; however, currently, there are no evidence-based treatment options available for this group. A cognitive therapy grounded in the principles of the Social Rank Theory, is being evaluated in terms of its effectiveness in reducing harmful compliance with command hallucinations.

**Methods/Design:**

This is a single blind, intention-to-treat, multi-centre, randomized controlled trial comparing Cognitive Therapy for Command Hallucinations + Treatment as Usual with Treatment as Usual alone. Eligible participants have to fulfil the following inclusion criteria: i) ≥16 years; ii) ICD-10 diagnosis of schizophrenia or related disorder; iii) command hallucinations for at least 6 months leading to risk of harm to self or others. Following the completion of baseline assessments, eligible participants will be randomly allocated to either the Cognitive Therapy for Command Hallucinations + Treatment as Usual group or the Treatment as Usual group. Outcome will be assessed at 9 and 18 months post randomization with assessors blind to treatment allocation. The primary outcome is compliance behaviour and secondary outcomes include beliefs about voices' power, distress, psychotic symptoms together with a health economic evaluation. Qualitative interviews with services users will explore the acceptability of Cognitive Therapy for Command Hallucinations.

**Discussion:**

Cognitive behaviour therapy is recommended for people with psychosis; however, its focus and evaluation has primarily revolved around the reduction of psychotic symptoms. In this trial, however, the focus of the cognitive behavioural intervention is on individuals' appraisals, behaviour and affect and not necessarily symptoms; this is also reflected in the outcome measures used. If successful, the results will mark a significant breakthrough in the evidence base for service users and clinicians and will provide a treatment option for this group where none currently exist. The trial will open the way for further breakthrough work with the 'high risk' population of individuals with psychosis, which we would intend to pursue.

**Trial registration:**

ISRCTN: ISRCTN62304114

## Background

Schizophrenia affects 0.8% of the UK population, usually starts in early adult life and leads to persistent disability in most cases [1]. It carries a high risk of suicide (8%) and deliberate self-harm [2] and, on a population basis, people with schizophrenia are more likely to perpetrate acts of aggression than their peers [3]. While drug treatment has improved, approaching fifty per cent will continue to experience treatment resistant symptoms [4] or symptoms arising from refusal to adhere with drug regimes [5]. Auditory hallucinations rank among the most prominent of the treatment resistant symptoms [6] and the most distressing and high risk of all are command hallucinations [6,7]. Command hallucinations are very prevalent in people who experience schizophrenia. A recent review by Shawyer et al [7] reported a median prevalence rate of 53% with a wide range from 18% to 89% in a sample of adult psychiatric patients. Furthermore, it was reported that 48% of command hallucinations stipulate harmful or dangerous actions [7] rising to 69% among patients in medium secure units [8]. This rate was significantly higher in the forensic population with 83% of voice hearers experiencing command hallucinations with *criminal *content [7].

However, the link between command hallucinations and harm to self or others is not straightforward. In the Macarthur study [9,10] no association was reported between the presence of delusions or command hallucinations and violence (GBH, assault and threats with a weapon). *Thoughts *about violence, on the other hand, were a strong predictor of violence six months later. A recent secondary analysis of the Macarthur study by Rogers [11,12] found that an additional significant predictor of aggression is beliefs about having to "*obey*" the voice. Thus, it appears to be the content of the individual's thinking and how this reflects the dynamics of the individual's relationship with their supposed persecutor who is commanding that is found to be predictive of harm to self and others in command hallucinations [13,14]. This was further confirmed by Trower et al [14] who found that it is the content of the voice and the individual's relationship with the personified voice that predicts compliance, distress and depression. These findings are in accordance with the social rank theory which suggests that individuals in subordinate positions will comply with the demands of those more dominant, or appease when compliance is risky or dangerous, but escape is impossible. In command hallucinations, the greater the power differential between the voice and the voice hearer, the greater the possibility of complying with "benevolent" voices or resisting but appeasing "malevolent" voices [13,14]. From the voice hearer's point of view, non-compliance risks harmful action from the voice (e.g. death to self or family), placing the individual in a dilemma often resolved by harmful appeasement or compliance. These findings have been independently replicated by Fox et al [15] comparing people who have complied with their voices vs. those who have resisted compliance. The former perceived their commanding voice to be more powerful and themselves to be inferior, hence motivating the need to submit to the voice and comply with its commands.

Nevertheless, predicting *who*, and *when *individuals will act on their voices has proven difficult in spite of these epidemiological data; also, *why *people respond to their voices in the above varying ways (e.g. complying, appeasing) is something that warrants exploring. The aim of our MRC COMMAND trial is to answer these questions while at the same time evaluate the efficacy of a cognitive behavioural therapy (CTCH) in reducing harmful compliance with command hallucinations. Cognitive behavioural therapy (CBT) is a psychological therapy originally developed for the management of emotional disorders like depression and anxiety disorders. The link between thinking and emotion/behaviour lies at the heart of this therapy such that emotional and behavioural responses are principally influenced by cognitive appraisals. CBT was further developed for the treatment of severe mental health problems like psychosis and has been thoroughly validated through large-scale pragmatic trials using primarily standard psychosis outcomes (e.g. Positive and Negative Symptom Scale) [16] (Birchwood & Trower, 2006). CBT is now recommended by the National Institute for Clinical Excellence [17] (2002) "to reduce psychotic symptoms, increase insight and promote medication adherence".

### Hypotheses

The primary hypothesis to be tested is whether in patients with command hallucinations who have acted on their voices and are therefore at high risk of doing so again, cognitive therapy for command hallucinations (CTCH) will prevent further harmful compliance behaviour, and thereby reduce risk.

Secondary hypotheses predict that:

(a) any reduction in compliance will be mediated by reduced conviction in the perceived power of the persecuting voice,

(b) CTCH will reduce delusional distress and depression, but

(c) we are not predicting any change in the frequency or topography of voices per se.

## Method/Design

The trial is funded by the Medical Research Council and has received ethical approval from the West Midlands Research Ethics Committee.

This is a single (rater) blind, prospective, pragmatic randomised controlled trial, using intention to treat comparing CTCH + TAU with TAU alone. The trial is recruiting participants with schizophrenia and schizoaffective disorders, with treatment resistant auditory hallucinations from inpatient wards and community mental health teams in Birmingham & Leicester, London and Manchester. Recruitment to the trial began in February 2008 and was completed in July 2010. Follow-up assessments began in November 2008 and will be completed in January 2012.

### The intervention

CTCH is designed to weaken and change beliefs about voices' power, thus enabling the individual to break free of the need to comply or appease and thereby reduce harmful compliance behaviour and distress. CTCH uses cognitive behavioural therapy to assess and modify conviction in four beliefs linked to the construct of voice power: that the voice has absolute *power *and *control*; that the individual must *comply *or *appease *or be severely punished; the *identity *of the voice (e.g. the Devil) and the *meaning *attached to the voice experience (e.g. the individual is being punished for past bad behaviour).

The CTCH protocol is described in Byrne et al [18], and in our casebook manual [19]. While the intervention is protocol based, it recognises individual differences in voice content, beliefs about voices and compliance. CTCH differs from previous and generic types and models of CBT for psychosis. First, it is informed by a well validated theoretical framework that predicts individuals' compliance with voices and the associated distress rather than the presence of symptoms per se. Second, it adheres to a staged process informed by the explanatory model. Third, the model proposes a single variable that is the target of therapy and also the hypothesised mediator: the power differential between voice and voice hearer.

The intervention is delivered in each centre by accredited cognitive therapists and clinical psychologists supervised by a lead clinician with expertise in CBT for psychosis. Group supervision across sites via videoconference was also conducted once a fortnight in order to monitor compliance to protocol and minimise centre differences in implementation. Adherence to protocol is monitored using our adapted version of the Cognitive Therapy Checklist [20].

CTCH is administered over a maximum period of 9 months. This includes a therapeutic window of approximately 25 sessions.

### Treatment as Usual (TAU)

Treatment as Usual (TAU) is provided by Community Mental Health or Assertive Outreach Teams, or inpatient ward teams. TAU including neuroleptic medication will be documented in line with the trial protocol derived from out pilot study [14].

### Inclusion and exclusion criteria

Eligible participants fulfil the following criteria: i) ICD-10 schizophrenia, schizoaffective (F20,22,23,25,28,29: WHO, 1999) [21], or ICD-10 diagnosis of Mood disorders (F32) under care of the clinical team (ii) age ≥16, (iii) history of command hallucinations of at least 6 months with history of harm to self, others or major social transgressions as a result of the commands; or harmful command hallucinations where the individual is distressed and appeasing the powerful voice. Exclusion criteria include: organic impairment or addictive disorder considered to be the primary diagnosis and insufficient command of the English language.

### Recruitment and randomisation

Eligible participants are identified by Clinical Studies Officers from the UK Mental Health Research Network who review the medical records looking for i) history of auditory hallucinations and ii) evidence of risky, aggressive and violent behaviour. A screening interview is conducted in order to confirm eligibility for the trial. Following the screening interview, eligible participants are invited to take part and asked to provide informed consent. Once informed consent has been obtained, trained researchers administer a battery of assessments and upon completion of the assessments, participants are randomly allocated either to the CTCH+TAU group or the TAU group (i.e. the Control group). Randomisation is conducted by OpenCDMS (http://www.opencdms.org) an online system managed by the University of Manchester in order to ensure concealment of group allocation. Randomisation was stratified by Centre using randomised-permuted blocks with a randomly-varying block size. Group allocation is revealed to the service user, clinician, trial manager, trial administrator and the trial therapists.

### Measures

#### Behavioural responses to voices

The level of compliance/resistance with each command is assessed using the Voice Compliance Scale (VCS) [13,14] after: a) conducting a thorough interview using the Cognitive Assessment of Voices schedule in order to obtain a detailed description of all voices as well as emotional and behavioural responses towards these voices b) interviewing and using information from other informants (carers, care-coordinator). Information collected by all these sources is collated in the form of a vignette and each behaviour is then classified as: neither appeasement nor compliant (1), symbolic appeasement, i.e. compliant with innocuous and/or harmless commands (2), actual appeasement i.e. preparatory acts or gestures (3), partial compliance with at least one severe command (4), full compliance with at least one severe command (5). These behaviours will be independently rated by the trial manager to ensure reliability of ratings.

#### Beliefs about voices

The Beliefs about Voices Questionnaire (BAVQ-R) [22] is used to assess key beliefs about the voices including benevolence/malevolence as well as emotional and behavioural reactions towards the voices. The scale has good test-retest (0.89) internal reliability (Cronbach's alpha = 0.85).

#### Power

The Voice Power Differential Scale (VPD) [23,24] is used to measure the perceived power differential between voice and voice hearer and includes the following constructs: strength, confidence, respect, ability to inflict harm, superiority and knowledge. This scale has good internal reliability (Cronbach's alpha = 0.85) with one week re-test reliability (r = 0.82).

#### Omniscience

The Personal Knowledge questionnaire/Omniscience scale [23] measures the voice hearer's beliefs about the voice's knowledge regarding personal information (e.g. "*The voice knows everything about me and my past"*).

#### Distress

Distress associated with voices is assessed using the Psychotic Symptoms Rating [25] Scales and specifically the section about auditory hallucinations (AH). The scale benefits from excellent psychometric properties with inter-rater reliability for the AH section ranging between 0.78-1.0.

#### Depression

The Calgary Depression Rating Scale for Schizophrenia [26] is a nine item observer rated measure specifically designed for schizophrenia, minimizing contamination by negative symptoms and the extrapyramidal side effects of neuroleptics. It is strongly correlated with the BDI [27] (r = 0.91) and is responsive to change in psychosis [14]. Recent studies have also supported the use of CDSS in healthy and non-psychotic populations [28].

#### Psychotic Symptoms

The *Positive and Negative Syndrome Scale *(PANSS) [29] includes scales of positive symptoms, negative symptoms and general psychopathology and is used widely in schizophrenia research.

#### Hopelessness & Suicidal Ideation

The *Beck Hopelessness Scale *[30] is used to assess three aspects of hopelessness: feelings about the future, loss of motivation and expectations. The *Beck Scale for Suicidal Ideation *[31] allows for a thorough examination of suicidal intent.

#### Childhood Trauma

The Childhood Trauma Questionnaire [32] is a 28-item self-report inventory measuring retrospectively experiences of childhood abuse and neglect. It consists of five subscales: emotional abuse, physical abuse, sexual abuse, emotional neglect and physical neglect, reflecting therefore a broad range of early adverse experiences. There is an additional 3-item Minimisation/Denial scale aiming to detect false-negative trauma reports [32]. Participants are asked to rate the frequency with which they have shared the reported childhood experiences on a 5-point Likert scale (1-5). The Childhood Trauma Questionnaire has been established as a reliable and valid measure of childhood traumatic experiences [32]. The CTQ has proved to have high internal reliability with *a *value ranging from 0.66 for the physical abuse subscale to 0.92 for the sexual abuse subscale. It has also demonstrated good test-retest reliability ranging from 0.79 to 0.86 for the five subscales over an average period of four months.

#### Health status

The EuroQol [33] is used to describe and evaluate health-related quality of life. Participants are requested (i) to rate their own health state of five dimensions (mobility, self-care, usual activities, pain/discomfort and anxiety/depression), (ii) to rate their current health status on a thermometer ranging 0-100 and (iii) to provide some background information on age, education, qualification, employment etc.

#### Health service costs

Costs of health and social care will be derived from data collected on each participant on inpatient/outpatient care, community and primary care services and the criminal justice system using the Economic Patient Questionnaire which includes a Psychiatric and a non-Psychiatric Hospital Record. The measure of patient outcome for the primary economic analysis will be quality adjusted life years (QALYs) gained at 2 years following recruitment to the trial. The QALYs will be estimated as the number of life years multiplied by the utility of each year of observed survival. The main framework of analysis will be cost effectiveness analysis and cost acceptability analysis.

### Primary Outcome

Primary outcome is compliance behaviour assessed using the Voice Compliance Scale (VCS) [13,14].

### Secondary outcomes

Secondary outcomes include beliefs about voices' power, distress, symptoms and costs. The acceptability of CTCH and user views about the active ingredients is explored through semi-structured interviews with a purposeful sample of users at the end of the intervention. The interview schedule will be piloted and modified as necessary. In all cases, interviews will be audio taped and fully transcribed. Interviews and analysis will be carried out concurrently and interviews will continue until data saturation is complete (approximately 10 users in each site). Data from semi-structured interviews will be analysed using a grounded theory approach [34] where theories are generated from data; this will follow the methodology of a recent study conducted by one of the applicants [35].

### Predictor variables

History of suicide, self harm and harm to others is collected as part of the baseline assessments. Data about childhood trauma and abuse is also routinely collected using the Childhood Trauma Questionnaire.

### Analysis

#### Power

A sample size of 100 per group will have 84% power to detect an absolute difference between a proportion who have acted on their voices of 40% under TAU and 20% under the CTCH arms using a Pearson chi-square test with two-tailed significance level of 0.05.

#### Planned Analyses

The evaluation of the primary outcome will be through an intention-to-treat (ITT) analysis using a logistic regression, allowing for centre membership and severity of command hallucinations at baseline. This will comprise a likelihood-based analysis to simultaneously estimate treatment effects at both the 9- and 18-month follow-up assessments and to allow for missing follow-up data that are assumed to be Missing at Random (MAR) in the terminology of Little & Rubin [36]. Analogous ANCOVA methods will be used for the analysis of the secondary outcomes.

#### Planned Subgroup Analyses

Predictors of treatment adherence will be investigated through the use of logistic regression modelling. In line with the pilot, this will include an analysis of the effect of ICD-10 classification (schizophrenia vs other psychoses) on treatment response. The effect of treatment on those who received it will be calculated through the estimation of a Complier-Average Causal Effect (CACE), using methods similar to those recently described by Dunn et al [37], including checking the sensitivity of the estimates to different operational definitions of adherence and assumptions concerning potentially missing outcome data. This approach will also be extended to test for and estimate the strength of possible mediating effects of therapy [38]. In a further exploratory analysis, predictors of command compliance, including self-harm and harm to others will also be identified through the use of logistic regression.

## Discussion

Preliminary findings [14] point towards evidence for the reasonable effectiveness of the CTCH but this large scale trial will provide more definitive results about the efficacy of the intervention and the durability of its effects. This intervention is among the very few that focus on targeting individuals' appraisals, behaviour and affect and not necessarily symptoms; this is also reflected in the outcome measures used.

If successful, the results will mark a significant breakthrough in the evidence base for clinicians and service users who act on their voices with harmful consequences or are at high risk of doing so.

## Competing interests

The authors declare that they have no competing interests. The trial is funded by the Medical Research Council (MRC) and the Department of Health.

## Authors' contributions

All authors participated in the design of the trial and read and approved the final manuscript

## Pre-publication history

The pre-publication history for this paper can be accessed here:

http://www.biomedcentral.com/1471-244X/11/155/prepub
